# Real-World Treatments and Clinical Outcomes in Advanced NSCLC without Actionable Mutations after Introduction of Immunotherapy in Japan

**DOI:** 10.3390/cancers14122846

**Published:** 2022-06-09

**Authors:** Hiroshi Nokihara, Takashi Kijima, Toshihide Yokoyama, Hiroshi Kagamu, Takuji Suzuki, Masahide Mori, Melissa L. Santorelli, Kazuko Taniguchi, Tetsu Kamitani, Masato Irisawa, Kingo Kanda, Machiko Abe, Thomas Burke, Yasushi Goto

**Affiliations:** 1 Department of Respiratory Medicine and Rheumatology, Graduate School of Biomedical Sciences, Tokushima University, 3-18-15 Kuramoto-cho, Tokushima 770-8503, Japan; 2 Department of Respiratory Medicine and Hematology, Hyogo College of Medicine, 1-1 Mukogawa-cho, Nishinomiya 663-8501, Japan; tkijima@hyo-med.ac.jp; 3 Department of Respiratory Medicine, Kurashiki Central Hospital, 1-1-1 Miwa, Kurashiki 710-8602, Japan; ty14401@kchnet.or.jp; 4 Department of Respiratory Medicine, Saitama Medical University International Medical Center, 1397-1 Yamane, Hidaka 350-1298, Japan; kagamu19@saitama-med.ac.jp; 5 Department of Respirology, Graduate School of Medicine, Chiba University, 1-8-1 Inohana, Chuo-ku, Chiba 260-8670, Japan; suzutaku@chiba-u.jp; 6 Department of Thoracic Oncology, National Hospital Organization, Osaka Toneyama Medical Center, 5-1-1 Toneyama, Toyonaka 560-8552, Japan; mori.masahide.fr@mail.hosp.go.jp; 7 Center for Observational & Real World Evidence (CORE), Merck & Co., Inc., 126 East Lincoln Ave., P.O. Box 2000, Rahway, NJ 07065, USA; melissa.santorelli@merck.com (M.L.S.); thomas_burke2@merck.com (T.B.); 8 MSD K.K., Kitanomaru Square, 1-13-12 Kudan-kita, Chiyoda-ku, Tokyo 102-8667, Japan; kazuko.taniguchi3@merck.com (K.T.); tetsu.kamitani@merck.com (T.K.); masato.irisawa@merck.com (M.I.); kingo.kanda@merck.com (K.K.); machiko.abe@merck.com (M.A.); 9 Department of Thoracic Oncology, National Cancer Center Hospital, 5-1-1 Tsukiji, Chuo-ku, Tokyo 104-0045, Japan; ygoto@ncc.go.jp

**Keywords:** chemotherapy, immune checkpoint inhibitor, non-small-cell lung cancer, nonplatinum therapy, overall survival

## Abstract

**Simple Summary:**

The aim of this study was to evaluate treatment patterns and real-world clinical outcomes since immunotherapy was introduced in Japan as the initial (first-line) therapy for treating patients with lung cancer, the leading cause of cancer-related deaths in Japan. For 1182 patients with advanced non-small-cell lung cancer, the survival rate at two years after starting first-line therapy was 40% with platinum doublet chemotherapy, 58% with immunotherapy, and 31% with nonplatinum regimens. The results of this large study enabled us to describe the characteristics of a real-world patient population, together with the treatment patterns for advanced non-small-cell lung cancer and clinical outcomes from real-world settings, where most patients receive treatment. Most first-line therapies were administered in accordance with contemporaneous national treatment guidelines, and the study findings indicate improvement in real-world clinical outcomes for patients with advanced non-small-cell lung cancer since the introduction of first-line immunotherapy.

**Abstract:**

The aims of this study were to describe systemic treatment patterns and clinical outcomes for unresectable advanced/metastatic non-small-cell lung cancer (NSCLC) by first-line regimen type in real-world clinical settings in Japan after the introduction of first-line immune checkpoint inhibitor (ICI) monotherapy in 2017. Using retrospective chart review at 23 study sites, we identified patients ≥20 years old initiating first-line systemic therapy from 1 July 2017 to 20 December 2018, for unresectable stage IIIB/C or IV NSCLC; the data cutoff was 30 September 2019. Eligible patients had recorded programmed death-ligand 1 (PD-L1) tumor proportion score (TPS) and no known actionable *EGFR*/*ALK*/*ROS1*/*BRAF* genomic alteration. Kaplan-Meier method was used to determine time-to-event endpoints. Of 1208 patients, 647 patients (54%) received platinum doublet, 463 (38%) received ICI monotherapy, and 98 (8%) received nonplatinum cytotoxic regimen as first-line therapy. PD-L1 TPS was ≥50%, 1–49% and <1% for 44%, 30%, and 25% of patients, respectively. Most patients with PD-L1 TPS ≥50% received ICI monotherapy (453/529; 86%). Excluding 26 patients with ECOG performance status of 3–4 from outcome analyses, the median patient follow-up was 11.3 months. With first-line platinum doublet, ICI monotherapy, and nonplatinum cytotoxic regimens, median overall survival (OS) was 16.3 months (95% CI, 14.0–20.1 months), not reached, and 14.4 months (95% CI, 10.3–21.2 months), respectively; 24-month OS was 40%, 58%, and 31%, respectively. Differences in OS relative to historical cohort data reported in Japan are consistent with improvement over time in real-world clinical outcomes for advanced NSCLC.

## 1. Introduction

Lung cancer remains one of the leading causes of new cancer cases and the number one cause of cancer-related deaths in Japan [1]. GLOBOCAN projections for Japan in 2020 included 138,532 new cases of lung cancer and 82,369 deaths from lung cancer, representing approximately 20% of all cancer-related deaths during the year [2]. Recent data from population-based registries in Japan indicate that about 38% of lung cancers are diagnosed at an advanced clinical stage when the 5-year relative survival falls below 7% [3,4]. Non-small-cell lung cancer (NSCLC) accounts for most cases of lung cancer both globally and in Japan [5,6].

The Japan Lung Cancer Society (JLCS) Guidelines for advanced NSCLC currently outline a personalized approach to treatment based on histology, performance status, age (<75 or ≥75 years), results of programmed death-ligand 1 (PD-L1) testing, and whether actionable genomic alterations are present [7,8,9]. Platinum-based chemotherapy was the standard first-line systemic anticancer therapy administered in Japan for patients with advanced NSCLC, in line with treatment guidelines, through 2015 [10,11], when national guidelines recommended platinum-based chemotherapy for patients <75 years and nonplatinum-based chemotherapy for those ≥75 years old with previously untreated advanced NSCLC negative for *EGFR* or *ALK* genomic alterations [7].

In December 2016, pembrolizumab monotherapy was approved, and in February 2017 reimbursed, as the first immune checkpoint inhibitor (ICI) of programmed death 1 (PD-1)/PD-L1 available in Japan for advanced NSCLC in the first-line setting. This initial first-line NSCLC approval was for pembrolizumab monotherapy of unresectable advanced/metastatic NSCLC with PD-L1 tumor proportion score (TPS) ≥50%. Other ICIs approved in Japan were for monotherapy of previously treated, unresectable advanced/metastatic NSCLC regardless of PD-L1 expression, including nivolumab monotherapy (December 2015) and, later, atezolizumab monotherapy (April 2018). Subsequent approvals have included expanded ICI monotherapy indications, along with ICI combination regimens with chemotherapy.

The recommendations in treatment guidelines are based on the results of randomized controlled trials, excluding patients with poor performance status and select comorbidities that may affect the efficacy and/or safety outcomes [12,13]. By contrast, patient populations treated in the less-controlled environment of real-world oncology settings tend to be more heterogeneous, older, and with worse performance status than those in clinical trials [5,11].

Prior observational studies have described systemic anticancer therapies administered to patients with advanced/metastatic NSCLC in the years from 2008 through 2015 in Japan [10,11,14]. However, information is limited about treatment patterns, use of PD-L1 testing, and real-world clinical outcomes since first-line ICI monotherapy approval in Japan. The aims of this study were to describe treatment patterns and clinical outcomes for advanced NSCLC without actionable mutations by regimen type in real-world clinical settings in Japan after the introduction of first-line pembrolizumab monotherapy and before subsequent approval of first-line ICI-chemotherapy combinations in December 2018.

## 2. Materials and Methods

### 2.1. Study Design and Patients

This retrospective observational study was conducted at 23 participating hospitals and medical centers throughout Japan. Data were abstracted retrospectively from medical records collected and maintained during routine clinical care. An electronic case report form was used by trained chart abstractors and investigators for the data collection process. Chart abstraction ran from 21 November 2019 to 29 May 2020. There were no changes to study procedures or analyses because of the COVID-19 pandemic other than remote source document verification when on-site visits were not allowed.

Patients ≥20 years old at diagnosis of pathologically confirmed, unresectable stage IIIB/C or IV NSCLC, staged per local guidelines [7], including those with initial diagnosis at an earlier stage of disease who experienced recurrence or progression, were eligible if initiating first-line systemic anticancer therapy for advanced NSCLC from 1 July 2017 to 20 December 2018 (index period). We required a record of tumor PD-L1 test results documented on or before the start of first-line therapy (index date). Patients who received first-line therapy as a clinical trial participant, who could be treated with curative intent through surgery or chemoradiation, and those with known actionable genomic alterations on/before the start of therapy, were excluded. Chart abstractors referred to a list of drug approval and reimbursement dates in Japan to identify actionable genomic alterations, as per the Japan Pharmaceuticals and Medical Devices Agency, including *EGFR* sensitizing mutations, *ALK* and *ROS1* gene rearrangements, and *BRAF* mutations [15]. Patients with incomplete medical records, such as those seen only for a consultation, were also excluded.

Data cutoff was on 30 September 2019, thus enabling a minimum of nine months potential follow-up from first-line therapy initiation. Patient follow-up ended at data cutoff, death, or when continued follow-up was no longer expected in the medical chart, whichever occurred first.

The study protocol conformed to the provisions of the Declaration of Helsinki and was approved by the local Ethics Committee at each participating center. Informed consent from individual patients was waived for this study by all Ethics Committees, as per applicable local laws, regulations, and guidelines for noninterventional research [16].

### 2.2. Assessments

The primary study objectives were to describe real-world treatment patterns and clinical outcomes in advanced NSCLC by line of therapy and regimen type, described here from first-line therapy initiation. For all eligible patients, we identified the first-line treatment regimens, which we then classified using three main categories (platinum-based doublet chemotherapy, ICI monotherapy, and nonplatinum cytotoxic regimens). An anti-vascular endothelial growth factor (anti-VEGF) agent, such as bevacizumab, was included when administered together with other systemic anticancer agent (s) (per drug label) and was thus grouped in the first-line platinum doublet or nonplatinum category, depending on how administered. Clinical outcomes from first-line therapy initiation (index date) were determined, after excluding patients with Eastern Cooperative Oncology Group (ECOG) performance status (PS) of 3–4 from primary outcome analyses because systemic therapy is not recommended in JLCS clinical guidelines for this patient population [7].

Overall survival (OS) was defined as the time from the index date until death from any cause, with censoring at the date of last clinical contact for patients who were still alive. We named the other study endpoints with the preceding “rw” (real-world) to distinguish them from the analogous, but not identical, endpoints that are determined prospectively in clinical trials. Real-world progression-free survival (rwPFS) associated with the first line of therapy was determined from the index date to the first documented (clinical or radiological) disease progression or death (whichever occurred first), with censoring at the start of a new line of therapy or date of last known activity for those with no new line of therapy. We defined the tumor response rate (rwTRR) as the proportion of patients who had radiologically documented or clinician-assessed best response of complete response (CR) or partial response (PR), and for these patients, we determined the duration of response (rwDOR) from the first record of CR or PR until the date of documented disease progression or death from any cause, whichever occurred first, with censoring of those with no documented disease progression or death at the start of a new line of therapy or date of last known activity for those with no new line of therapy. The disease control rate (rwDCR) was defined as the proportion of patients with radiologically documented or clinician-assessed best response of CR, PR, or stable disease.

We determined the time on treatment (rwToT) as the length of time between the first and last administration dates of the first-line regimens. Also known as real-world time to treatment discontinuation, rwToT is associated at the patient-level with PFS and OS in clinical trials and real-world data for continuously administered therapies, such as ICIs [17,18,19,20,21]. Therapy was considered discontinued at the last dose if patients died, continued to the next line of therapy, or had a gap ≥120 days between their last dose and last known activity in the dataset; all other patients were censored at their last first-line therapy administration date. We also determined the time to next treatment (rwTTNT), defined as the length of time from the index date to the date of subsequent (second-line) therapy initiation, with censoring at the last known activity date if no subsequent treatment was received. In addition, we described treatment sequences from first- through third-line therapy.

As an exploratory clinical analysis, we determined rwPFS on the next line of therapy (rwPFS2) for all patients who received ICI monotherapy, including those with ECOG PS of 3–4, for a better understanding of disease progression beyond first-line. We defined rwPFS2 as the time from first-line therapy initiation until documented (clinical or radiological) disease progression while on second-line therapy or death, whichever occurred first. Patients without documented disease progression or death were censored for rwPFS2 on the last day of follow-up or last assessment date (further details are in the online Supplementary Methods).

### 2.3. Statistical Analysis

Descriptive statistics were used to summarize patient characteristics and treatment regimens by the line of therapy. Time-to-event analyses were performed using the Kaplan-Meier method to estimate medians with 95% confidence intervals (CI) overall and by first-line regimen for OS and rwPFS and by first-line regimen for rwDoR, rwTTNT, rwToT, and rwPFS2. Landmark analyses were performed at prespecified timepoints, as defined for each outcome and including primary subgroup analyses by histology (nonsquamous and squamous), age group (<75 and ≥75 years), baseline ECOG PS (0–1 and 2), and PD-L1 TPS (≥50%, 1–49%, <1%).

The last known activity date for censoring was defined analytically for each patient as the latest date registered in the database among the dates of treatments and other health care resource use (hospitalizations, emergency room visits, outpatient visits, outpatient procedures, rebiopsy for molecular testing, laboratory testing, imaging, concomitant medication use). Handling of missing data is described in Appendix A.

The Clopper Pearson exact method was used to calculate 95% CIs for prevalence, and Poisson distribution was used to calculate 95% CIs for incidence.

Analyses were prespecified before the database lock in the final statistical analysis plan. Sample size calculations were not performed as this study was descriptive with no hypothesis testing. Analyses were performed using SAS software, version 9.4 or later (SAS Institute, Cary, NC, USA).

## 3. Results

### 3.1. Patients and First-Line Treatment Patterns

A total of 1208 eligible patients with locally advanced or metastatic NSCLC were identified at 23 participating clinical centers in Japan. The median patient age was 70 years (range, 27–92 years), and 975 patients (81%) were men, and 90% of patients were current or former smokers (Table 1). Slightly over half of patients (648; 54%) had ECOG PS of 0 or 1, 93 (8%) had PS of 2, and 26 (2%) had PS of 3 or 4; 441 patients (37%) had unknown PS.

The initial diagnosis of NSCLC was made at an advanced stage for 80% of patients; and, overall, 62% of tumors were nonsquamous, 32% were squamous, and 7% were of unknown histology (Table 1). The baseline PD-L1 TPS was ≥50%, 1–49% and <1% for 529 (44%), 367 (30%), and 302 patients (25%), respectively; PD-L1 TPS was not evaluable for 10 patients (1%). Most patients had a test for *EGFR* mutations (895; 74%), and 809 (67%) were tested for *ALK* rearrangements, 450 (37%) for *ROS1* rearrangements, and 11 (1%) for *BRAF* mutations.

In first-line therapy, 647 patients (54%) were treated with platinum doublet chemotherapy, 463 (38%) with ICI monotherapy, and 98 (8%) with a nonplatinum cytotoxic regimen. The median ages of patients treated with platinum doublet and ICI monotherapy were similar (69 and 70 years, respectively), whereas the median age of those treated with a nonplatinum regimen was 80 years, and 81% of patients who received a nonplatinum regimen were ≥75 years old (Table 1).

The majority of the 529 patients with high PD-L1 expression (TPS ≥50%) received ICI monotherapy (453; 86%). Conversely, of the 463 patients who received ICI monotherapy, 453 (98%) had PD-L1 TPS ≥50%. First-line regimens are summarized in Supplementary Table S1 according to tumor PD-L1 expression and histology.

The two most common regimens for first-line platinum doublet therapy were carboplatin plus nab-paclitaxel (160; 25%) and carboplatin plus pemetrexed (120; 19%). Pembrolizumab was the most common ICI, administered to 459 of the 463 patients (99%) who received first-line ICI monotherapy; three patients received nivolumab, and one received atezolizumab.

### 3.2. Real-World Outcomes of Treatment and Subsequent Therapy, by First-Line Regimen

Of 1208 patients overall, 26 patients (2%) had ECOG PS of 3 or 4 and were excluded from primary clinical outcome analyses. The baseline characteristics of the remaining 1182 patients are summarized by treatment regimen in Supplementary Table S2. Median patient follow-up for these patients from first-line therapy initiation to the date of death, end of patient follow-up, or data cutoff, whichever occurred first, was 11.3 months (range, <0.1 to 26.9 months).

**Figure 1:**
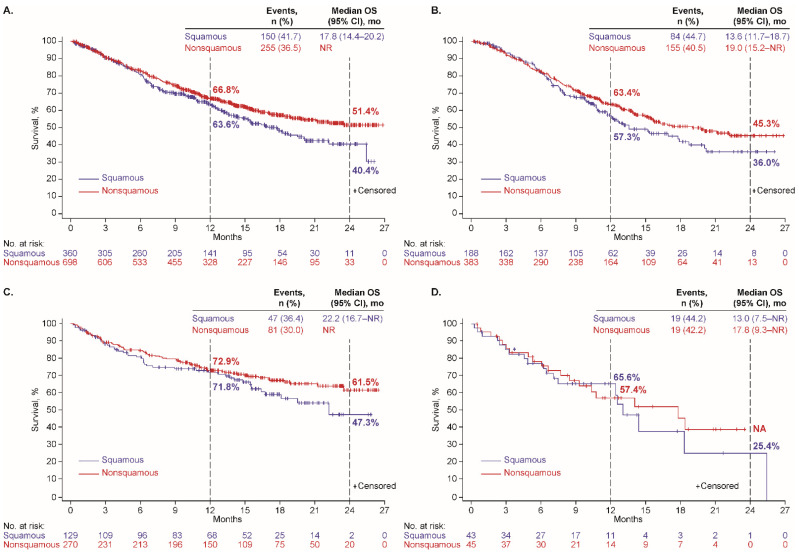
Overall survival by tumor histology among patients who received first-line therapy (**A**) overall and with (**B**) platinum doublet, (**C**) ICI monotherapy, and (**D**) nonplatinum cytotoxic regimen.

**Figure 2:**
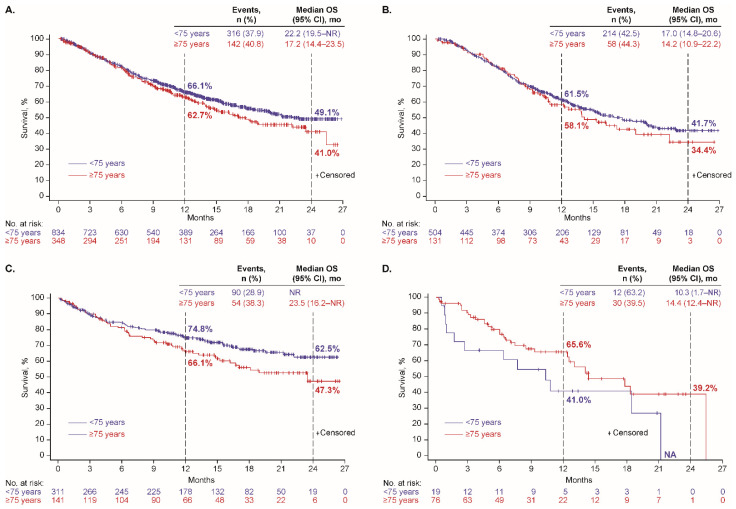
Overall survival by age group among patients who received first-line therapy (**A**) overall and with (**B**) platinum doublet, (**C**) ICI monotherapy, and (**D**) nonplatinum cytotoxic regimen.

At data cutoff, 458 patients (39%) had a recorded date of death, including 272/635 (43%), 144/452 (32%), and 42/95 (44%) who received first-line platinum doublet, ICI monotherapy, and nonplatinum cytotoxic regimens, respectively. The median OS was 16.3 months (95% CI, 14.0–20.1 months), not reached (NR), and 14.4 months (95% CI, 10.3–21.2 months), respectively; and Kaplan-Meier estimates of OS at 24 months were 40%, 58%, and 31%, respectively (Table 2). Kaplan-Meier plots of OS, overall and with each regimen, by histology (nonsquamous or squamous), are depicted in Figure 1, by age group (<75 or ≥75 years) in Figure 2, and by performance status (PS 0–1 or 2) in Supplementary Figure S1.

For patients who received platinum doublet therapy in the first line, the median rwPFS was 5.8 months (95% CI, 5.3–6.3), and the rwTRR was 29.6% (188 of 635 patients; 95% CI, 26.1–33.3). For the 182 patients evaluable for the duration of response, the median rwDoR was 5.6 months (95% CI, 4.8–6.0; Table 2). In the first-line ICI monotherapy cohort, median rwPFS was 9.7 months (95% CI, 8.1–11.1); rwTRR was 36.7% (166 of 452; 95% CI, 32.3–41.4); and median rwDoR was 16.0 months (95% CI, 12.9–NR) for the 164 evaluable patients. In the first-line nonplatinum cohort, median rwPFS was 4.9 months (95% CI, 3.5–5.7); rwTRR was 11.6% (11 of 95; 95% CI, 5.9–19.8); and median rwDoR was 4.0 months (95% CI, 1.7–NR) for the 11 evaluable patients. Kaplan-Meier plots of rwPFS by tumor histology, age group, and ECOG PS are depicted in Supplementary Figures S2–S4, respectively.

Treatment-related outcomes, including rwToT and rwTTNT, are summarized by first-line regimen in Supplementary Table S3. The median rwToT was 3.0 months (95% CI, 2.8–3.3) in the platinum doublet cohort, 5.5 months (95% CI, 4.4–6.7) in the ICI monotherapy cohort, and 2.2 months (95% CI, 1.2–3.2) in the nonplatinum regimen cohort. With first-line ICI monotherapy, on-treatment rates were 48.8% at 6 months, 25.6% at 12 months, and 12.1% at 18 months. Supplementary Figure S5 depicts rwToT overall and by histology, age group, and ECOG PS for patients treated with first-line ICI monotherapy.

The median rwTTNT was 6.3 months (95% CI, 5.9–6.9) in the platinum doublet cohort, 18.3 months (95% CI, 14.0–NR) in the ICI monotherapy cohort, and 6.7 months (95% CI, 4.3–9.4) in the nonplatinum regimen cohort. At 12 months, the Kaplan-Meier rates of patients who had not initiated a subsequent treatment line were 25%, 59%, and 34%, respectively (Supplementary Table S3).

A total of 616 patients (52%) continued to second-line therapy, most commonly ICI monotherapy (47%), and of the 616 patients, 278 (45%, or 24% overall) continued to third-line therapy, most commonly a nonplatinum cytotoxic regimen (65%; Table 3). Of the 635 patients who received first-line platinum doublet, 405 (64%) received second-line therapy and 190/405 (47%) continued to third-line therapy. Of the 452 patients who received first-line ICI monotherapy, 167 (37%) received second-line therapy and 77/167 (46%) continued to third-line therapy. Of the 95 patients who received a first-line nonplatinum regimen, 44 (46%) received second-line therapy and 11/44 (25%) continued to third-line therapy (details in Supplementary Table S4).

Among all 463 patients who were treated with first-line ICI monotherapy (including the 11 patients with ECOG PS 3–4 who were excluded from other outcomes analyses), 186 (40%) experienced a rwPFS2 event. The median rwPFS2 was 20.6 months (95% CI, 15.7–NR), and at 12 and 24 months, the Kaplan-Meier rwPFS2 rate was 63.2% (95% CI, 58.2–67.8) and 46.0% (95% CI, 39.4–52.3), respectively.

## 4. Discussion

The results of this large, retrospective chart review study, conducted after first-line ICI monotherapy for advanced NSCLC became available in Japan, indicate that most first-line therapies administered were in accordance with national treatment guidelines for the index period (July 2017 to December 2018) [7]. Of the 1208 patients studied, none with known actionable *EGFR*/*ALK*/*ROS1*/*BRAF* genomic alterations before initiating first-line therapy, 54% received platinum doublet chemotherapy, 38% ICI monotherapy, and 8% a nonplatinum cytotoxic regimen as their first systemic anticancer therapy. After excluding the 26 patients with ECOG PS of 3–4, the overall median OS was 21.1 months, and the Kaplan-Meier 24-month OS rate was 46.9%. For patients who received first-line platinum doublet-based chemotherapy, median OS was 16.3 months and the 24-month OS rate was 40.3%. We observed that median OS was not reached for the first-line ICI monotherapy cohort, which experienced a 24-month OS rate of 57.8%. For the minority of patients who received first-line nonplatinum cytotoxic regimens, the median OS was 14.4 months, and the 24-month OS rate was 31.1%.

Almost all patients (98%) who received first-line ICI monotherapy had high-expressing PD-L1 tumors (TPS ≥ 50%); conversely, the majority of patients (86%) with PD-L1 TPS ≥50% received first-line ICI monotherapy, per guidelines, rather than platinum doublet chemotherapy or a nonplatinum cytotoxic regimen. During the study period, the applicable Japanese lung cancer treatment guidelines (2016–17) designated chemotherapy as the standard first-line treatment option for patients with advanced NSCLC with PD-L1 TPS <50%, or unknown PD-L1 expression [7,8]. A greater percentage of older patients (≥75 years) and patients with PS of 2 received a nonplatinum cytotoxic regimen as the first-line regimen type relative to younger patients (<75 years) and patients with a more favorable performance status (PS 0–1), respectively, in line with guideline recommendations. First-line platinum doublet regimens were prescribed to 60% of patients <75 years of age and just 37% of patients ≥75 years of age.

For patients who received first-line ICI monotherapy, the 12- and 24-month OS rates of 72.1% and 57.8% in this study were consistent with 12- and 24-month OS rates of 70.3% and 51.5% in the KEYNOTE-024 clinical trial of first-line pembrolizumab monotherapy for metastatic NSCLC with PD-L1 TPS ≥50% [22]. Moreover, while we used the documentation in medical charts to capture disease progression for determining rwPFS associated with first-line therapy (censoring patients with no progression at the start of second-line therapy), we also determined rwPFS2 for all patients who received ICI monotherapy for a better understanding of disease progression beyond first-line. We found that the median rwPFS2 for all patients treated with first-line ICI monotherapy in the real-world setting of this study (20.6 months; 95% CI, 15.7–NR) resembled that observed in KEYNOTE-024 (24.1 months; 95% CI, 15.0 to 31.4) [23].

Overall, the clinical outcomes of patients included in this study suggest that outcomes for patients with advanced NSCLC in Japan may be improving over time. An analysis of phase III clinical trials published from 1998 to 2015 found a measurable, progressive increase in OS after first-line therapy for advanced NSCLC [24]. Similarly, the survival results in this study relative to historical cohort data reported in Japan, while not directly comparable, are consistent with improvement in real-world clinical outcomes over time. For example, a retrospective chart review study conducted in Japan at five clinical centers just before the introduction of ICI monotherapy for advanced NSCLC reported a median OS of 10.1 months (95% CI, 7.3–14.4) and 6.9 months (5.6–10.0) from the start of first-line therapy for patients with squamous and nonsquamous (*EGFR/ALK*-negative or unknown) advanced NSCLC, respectively [11]. In the large Japanese Lung Cancer Registry study of patients with lung cancer diagnosed in 2012, the 3-year survival rate was only 17% for those with NSCLC who received chemotherapy but no EGFR tyrosine kinase inhibitor, suggestive of no actionable genomic alterations as in the present study [5].

These and other prior large studies of treatment patterns and/or outcomes for unresectable/recurrent advanced NSCLC were conducted before the availability of immunotherapy in Japan [5,10]. More recent observational studies of NSCLC in Japan tend to be small and focused on single treatment types or patients with specific prognostic factors [25,26]. Instead, we were able to study a large number of patients (*n* = 1208) treated at 23 different oncology centers geographically well-distributed throughout Japan after first-line ICI monotherapy became available in February of 2017, and we included several categories of systemic anticancer regimens.

The results of this study describe the characteristics of this large real-world patient population, together with the treatment patterns for advanced NSCLC and outcomes from real-world settings, where most patients receive treatment. The full study population included 93 patients (8%) with ECOG PS of 2, 26 patients (2%) with ECOG PS of 3–4, plus an additional 441 patients (37%) for whom PS was not available on the retrospective chart review, in contrast to clinical trial populations, which are typically limited to patients with good performance status (ECOG 0–1) [27,28,29]. The median age of 70 years (with 30% of patients ≥75 years old) and the overall percentage of men (81%) were similar to real-world populations in prior observational studies [5,11], whereas the median age tends to be younger in clinical trials, both in Japan and elsewhere [27,28]. Other strengths of the study include the median follow-up of almost one year (11.3 months) after first-line therapy initiation and the geographical dispersion of study sites that was planned to approximate the population distribution in Japan to improve the generalizability of study results. In addition, the data were drawn from a manual review of medical charts and thus were not limited to structured electronic data. Finally, medical charts were assessed for patient eligibility sequentially from oldest to newest in an effort to reduce selection bias.

We acknowledge that the study was conducted at selected clinical centers and thus may not represent the entire patient population or treatment practices in Japan. Moreover, this study was descriptive in nature and was not designed as a comparative effectiveness study; therefore, causality inferences are not appropriate. The missing ECOG PS information for one-third of patients resulted in lower patient numbers for assessing OS and rwPFS according to baseline performance status. The tumor response data were collected as recorded in the medical charts and cannot be considered equivalent to Response Evaluation Criteria in Solid Tumors (RECIST 1.1) as applied in clinical trials [30]. It is also worth highlighting that differences in patient characteristics between regimen types could explain observed outcome differences among treatment cohorts.

Further study is needed on outcomes with combination ICI-chemotherapy, in line with more recent guidelines [31], in addition to longer follow-up to assess long-term clinical outcomes. Studies that include assessments of prognostic factors, patient-reported outcomes, and health care resource use are also needed. The present study has a planned prospective phase, enrolling patients from 14 November 2019 through December 2021, corresponding to the pembrolizumab monotherapy and combination therapy access period in Japan.

## 5. Conclusions

In alignment with the JLCS Guidelines, ICI monotherapy was the most commonly used first-line treatment for patients with advanced NSCLC with high PD-L1 expression and without actionable genomic alterations following the introduction of immunotherapy in Japan. Likewise, platinum doublet-based chemotherapy was the most common therapy for patients with PD-L1 TPS <50% and nonplatinum regimens were most commonly administered in the first line to older patients. The differences in OS relative to historical cohort data reported in Japan are consistent with an improvement in real-world clinical outcomes over time.

## Figures and Tables

**Table 1:** Baseline patient characteristics overall and by first-line systemic therapy regimen.

Characteristic	All Patients *n* = 1208	First-Line Treatment Regimen		
		Platinum Doublet *n* = 647	ICI Monotherapy *n* = 463	Nonplatinum Cytotoxic *n* = 98
**Men**	975 (80.7)	531 (82.1)	362 (78.2)	82 (83.7)
**Age**				
Median (range)	70 (27–92)	69 (27–83)	70 (30–89)	80 (45–92)
<75 years old	850 (70.4)	514 (79.4)	317 (68.5)	19 (19.4)
≥75 years old	358 (29.6)	133 (20.6)	146 (31.5)	79 (80.6)
**Smoking status, *n* ^a^**	1184	630	457	97
Current smoker	182 (15.4)	109 (17.3)	67 (14.7)	6 (6.2)
Former smoker	884 (74.7)	468 (74.3)	341 (74.6)	75 (77.3)
Never smoker	118 (10.0)	53 (8.4)	49 (10.7)	16 (16.5)
**ECOG performance status**				
PS 0–1	648 (53.6)	366 (56.6)	242 (52.3)	40 (40.8)
PS 2	93 (7.7)	38 (5.9)	38 (8.2)	17 (17.3)
PS 3–4	26 (2.2)	12 (1.9)	11 (2.4)	3 (3.1)
PS unknown	441 (36.5)	231 (35.7)	172 (37.1)	38 (38.8)
**Advanced NSCLC at diagnosis**	967 (80.0)	515 (79.6)	372 (80.3)	80 (81.6)
**NSCLC histology, *n* ^a^**	1155	621	440	94
Nonsquamous	712 (61.6)	391 (63.0)	275 (62.5)	46 (48.9)
Squamous	367 (31.8)	191 (30.8)	132 (30.0)	44 (46.8)
Other	76 (6.6)	39 (6.3)	33 (7.5)	4 (4.3)
**PD-L1 TPS ^b^**				
≥50%	529 (43.8)	70 (10.8)	453 (97.8)	6 (6.1)
1–49%	367 (30.4)	303 (46.8)	8 (1.7)	56 (57.1)
<1%	302 (25.0)	265 (41.0)	1 (0.2)	36 (36.7)
Not evaluable	10 (0.8)	9 (1.4)	1 (0.2)	0
**Brain metastasis**	202 (16.7)	103 (15.9)	93 (20.1)	6 (6.1)
Pretreated ^c^	130 (64.4)	59 (57.3)	67 (72.0)	4 (66.7)
**Liver metastasis**	98 (8.1)	51 (7.9)	37 (8.0)	10 (10.2)
**Bone metastasis**	306 (25.3)	167 (25.8)	125 (27.0)	14 (14.3)
**History of lung surgery**	204 (16.9)	122 (18.9)	67 (14.5)	15 (15.3)
**Prior radiation therapy**	98 (8.1)	48 (7.4)	42 (9.1)	8 (8.2)
**Prior chemoradiation**	39 (3.2)	19 (2.9)	18 (3.9)	2 (2.0)

Data are n (%) unless otherwise indicated. Percentages may not add up to 100% because of rounding. ^a^ Percentages are of known totals. ^b^ PD-L1 testing was conducted using the pembrolizumab companion diagnostic (PD-L1 IHC 22C3 pharmDx, Agilent Technologies, Carpinteria, CA, USA) for all but 4 patients (3 of whom had missing information). ^c^ Brain metastases treated before initiation of first-line therapy. ECOG, Eastern Cooperative Oncology Group; ICI, immune checkpoint inhibitor; PD-L1 TPS, programmed death-ligand 1 tumor proportion score.

**Table 2:** Real-world clinical outcomes by first-line treatment regimen.

	All Patients *n* = 1182	Platinum Doublet *n* = 635	ICI Monotherapy *n* = 452	Nonplatinum Cytotoxic *n* = 95
**Diagnosis to 1L, median (range), months**	0.9 (0–278.8)	0.9 (0–278.8)	1.0 (0–129.2)	1.0 (0–54.5)
**Overall survival (OS), *n***	1182	635	452	95
Events, *n* (%)	458 (38.7)	272 (42.8)	144 (31.9)	42 (44.2)
Median OS (95% CI), months	21.1 (18.3–NR)	16.3 (14.0–20.1)	NR	14.4 (10.3–21.2)
OS rate, % (95% CI)				
At 6 months	82.0 (79.6–84.1)	81.6 (78.3–84.5)	83.5 (79.6–86.7),	77.3 (67.0–84.7)
At 12 months	65.2 (62.2–68.0)	60.8 (56.6–64.8)	72.1 (67.5–76.2)	60.0 (48.0–70.0)
At 24 months	46.9 (42.6–51.1)	40.3 (34.4–46.1)	57.8 (50.9–64.2)	31.1 (16.4–47.1)
**Real-world PFS (rwPFS), *n***	1181	634	452	95
Events, *n* (%)	708 (59.9)	391 (61.7)	257 (56.9)	60 (63.2)
Median rwPFS (95% CI), months	6.4 (6.0–6.9)	5.8 (5.3–6.3)	9.7 (8.1–11.1)	4.9 (3.5–5.7)
rwPFS rate, % (95% CI)				
At 6 months	53.4 (50.3–56.4)	48.7 (44.3–53.0)	61.5 (56.8–66.0)	35.6 (24.6–46.7)
At 12 months	31.9 (28.8–34.9)	22.3 (18.3–26.4)	44.1 (39.1–48.9)	19.3 (10.4–30.2)
At 24 months	22.8 (19.5–26.3)	14.8 (10.8–19.4)	33.0 (27.6–38.5)	n/a
**rwTumor response, *n***	n/a ^a^	188	166	11
rwTRR, % (95% Clopper Pearson CI)	–	29.6 (26.1–33.3)	36.7 (32.3–41.4)	11.6 (5.9–19.8)
rwDisease control, *n*	n/a	339	243	45
rwDCR, % (95% Clopper Pearson CI)	–	53.4 (49.4–57.3)	53.8 (49.0–58.4)	47.4 (37.0–57.9)
rwDuration of response (rwDoR), *n*	n/a	182	164	11
Events, *n* (%)	–	107 (58.8)	68 (41.5)	8 (72.7)
Median rwDoR (95% CI), months	–	5.6 (4.8–6.0)	16.0 (12.9–NR)	4.0 (1.7–NR)

^a^ Tumor response was determined only for the first-line regimen. 1L, first-line therapy for advanced NSCLC; n/a, not assessed; DCR, disease control rate; NR, not reached; PFS, progression-free survival; rw, real-world; TRR, tumor response rate.

**Table 3:** Subsequent systemic therapy regimens by treatment line.

	All Patients *n* = 1182	First-Line Treatment Regimen		
		Platinum Doublet *n* = 635	ICI Monotherapy *n* = 452	Nonplatinum Cytotoxic *n* = 95
**Second-line regimen, *n* (%) ^a^**	616 (52.1)	405 (63.8)	167 (36.9)	44 (46.3)
Platinum doublet	163 (26.5)	37 (9.1)	126 (75.4)	0
ICI monotherapy	292 (47.4)	259 (64.0)	3 (1.8)	30 (68.2)
ICI-chemotherapy combination	1 (0.2)	0	1 (0.6)	0
Nonplatinum cytotoxic	158 (25.6)	108 (26.7)	36 (21.6)	14 (31.8)
Tyrosine kinase inhibitor	1 (0.2)	1 (0.2)	0	0
Other	1 (0.2)	0	1 (0.6)	0
**Third-line regimen, *n* (%) ^a^**	278 (23.5)	190 (29.9)	77 (17.0)	11 (11.6)
Platinum doublet	18 (6.5)	11 (5.8)	7 (9.1)	0
ICI monotherapy	75 (27.0)	59 (31.1)	14 (18.2)	2 (18.2)
Nonplatinum cytotoxic	181 65.1)	119 (62.6)	53 (68.8)	9 (81.8)
Tyrosine kinase inhibitor	2 (0.7)	0	2 (2.6)	0
Other	2 (0.7)	1 (0.5)	1 (1.3)	0

Drug regimens are shown as a percentage of the relevant treatment line. Percentages may not total 100 because of rounding. ^a^ Initiation of a new line of therapy was determined by investigators using information captured in the medical chart, together with their medical discretion, at the time of chart abstraction. ICI, immune checkpoint inhibitor.

## Data Availability

Patient medical record data are not publicly available and cannot be shared.

## Supplementary Materials

The following supporting information can be downloaded at: https://www.mdpi.com/article/10.3390/cancers14122846/s1, Table S1: First-line therapy regimens by PD-L1 expression and tumor histology; Table S2. Baseline characteristics of patients included in outcome analyses; Table S3: Real-world treatment outcomes for first-line regimens; Table S4: Treatment sequence overall and by tumor histology; Figure S1: Overall survival by ECOG performance status among patients who received first-line therapy (A) overall and with (B) platinum doublet, (C) ICI monotherapy, and (D) nonplatinum cytotoxic regimen; Figure S2: Real-world progression-free survival (rwPFS) by tumor histology among patients who received first-line therapy (A) overall and with (B) platinum doublet, (C) ICI monotherapy, and (D) nonplatinum cytotoxic regimen; Figure S3: Real-world progression-free survival (rwPFS) by age group among patients who received first-line therapy (A) overall and with (B) platinum doublet, (C) ICI monotherapy, and (D) nonplatinum cytotoxic regimen; Figure S4: Real-world progression-free survival (rwPFS) by ECOG PS among patients who received first-line therapy (A) overall and with (B) platinum doublet, (C) ICI monotherapy, and (D) nonplatinum cytotoxic regimen; Figure S5: Real-world time on treatment for patients who received first-line ICI monotherapy (A) overall; (B) by histology, (C) by age group, and (D) by ECOG PS.

## Appendix A. Handling of Missing Data

Partial dates collected as such in electronic case report forms were handled as follows:

- If day of start date was missing, it was imputed to the first day of the month.
- If day of stop date was missing, it was imputed to the last day of the month, death date, or date of withdrawal, whichever was earlier.
- If either the month or the year elements of the event date were missing, then the date was not imputed and was assigned a missing value.

Missing data on treatment partial start date/stop date were handled as follows:

- If a stop date for a treatment was missing, the start date for the subsequent treatment was used to impute the stop date using this formula: the stop date of a treatment = the start date of the next treatment − 1 day. If a patient was indicated as continuing in the treatment at the study end, the study end date was used to impute the stop date. If the stop date for the last treatment was missing and the patient died before the completion of the study, then the stop date was imputed as date of death.
- If a start date for a treatment was missing, the stop date for the previous treatment was used to impute the start date using this formula: the start date of a treatment = the stop date of the last treatment + 1 day.
- If these imputation rules resulted in illogical (negative) time-to-event durations, the negative durations were replaced with 1 day. This may have occurred, for example, if death date was imputed to the first of the month, whereas the patient started their next line of treatment (based on drug prescription dates) after this imputed date.
