# Supplementary material for: Real-World Treatments and Clinical Outcomes in Advanced NSCLC without Actionable Mutations after Introduction of Immunotherapy in Japan
Source: Cancers (Basel). 2022 Jun 9;14(12):2846. doi: 10.3390/cancers14122846 (PMC9220782; doi:10.3390/cancers14122846)
Supplement: Supplementary file 1 [file cancers-14-02846-s001.zip › cancers-1743170-supplementary.pdf]

Online supplement

Real-World Treatments and Clinical Outcomes in Advanced NSCLC without Actionable Mutations after Introduction of Immunotherapy in Japan

Hiroshi Nokihara, Takashi Kijima, Toshihide Yokoyama, Hiroshi Kagamu, Takuji Suzuki, Masahide Mori, Melissa L. Santorelli, Kazuko Taniguchi, Tetsu Kamitani, Masato Irisawa, Kingo Kanda, Machiko Abe, Thomas Burke and Yasushi Goto

Supplemental Methods

The full definition and censoring rules for rwPFS2 were as follows:

- Real-world progression-free survival on next line therapy (rwPFS2) was defined as the time from first dose of first-line (1L) treatment to documented (earliest of clinical or radiological) disease progression while on next-line treatment, i.e., second-line (2L) therapy, or death due to any cause, whichever occurred first. Patients without documented disease progression or death were censored for rwPFS2 on the last day of follow-up or last assessment date. The last day of follow-up or last assessment date was operationalized as last known activity. Patients who did not receive 2L therapy or who stopped 2L therapy without progressive disease and did not start third-line (3L) therapy were (a) counted as an event at the time of death if the patient died, or (b) censored at the time of last known survival if the patient was alive. Patients who stopped 2L therapy with progressive disease were counted as an event at the time of progression, and patients who stopped 2L therapy without progression and started 3L therapy were counted as an event at the start of 3L therapy."

Table S1. First-line therapy regimens by PD-L1 expression and tumor histology.

PD-L1 TPS	Platinum Doublet		ICI Monotherapy		Nonplatinum Cytotoxic	
	Nonsquamous *n* = 391	Squamous *n* = 191	Nonsquamous *n* = 275	Squamous *n* = 132	Nonsquamous *n* = 46	Squamous *n* = 44
≥50%	33 (8.4)	29 (15.2)	272 (98.9)	127 (96.2)	3 (6.5)	1 (2.3)
1–49%	172 (44.0)	99 (51.8)	3 (1.1)	5 (3.8)	24 (52.2)	31 (70.5)
<1%	181 (46.3)	63 (33.0)	0	0	19 (41.3)	12 (27.3)
Not evaluable	5 (1.3)	0	0	0	0	0

1L, first-line; ICI, immune checkpoint inhibitor; PD-L1, programmed death-ligand 1; TPS, tumor proportion score.

Table S2. Baseline characteristics of patients included in outcome analyses.

Characteristic	All Patients *n* = 1182	First-Line Regimen		
		Platinum *n* = 635	ICI Monotherapy *n* = 452	Nonplatinum *n* = 95
**Men**	954 (80.7)	522 (82.2)	353 (78.1)	79 (83.2)
**Age**				
Median (range)	70 (27–92)	69 (27–83)	70 (30–89)	80 (45–92)
<75 years old	834 (70.6)	504 (79.4)	311 (68.8)	19 (20.0)
≥75 years old	348 (29.4)	131 (20.6)	141 (31.2)	76 (80.0)
**Smoking status, *n*** [a]	1158	618	446	94
Current smoker	178 (15.4)	106 (17.2)	66 (14.8)	6 (6.4)
Former smoker	866 (74.8)	461 (74.6)	332 (74.4)	73 (77.7)
Never smoker	114 (9.8)	51 (8.3)	48 (10.8)	15 (16.0)
**ECOG PS**				
PS 0–1	648 (54.8)	366 (57.6)	242 (53.5)	40 (42.1)
PS 2	93 (7.9)	38 (6.0)	38 (8.4)	17 (17.9)
PS unknown	441 (37.3)	231 (36.4)	172 (38.1)	38 (40.0)

**Advanced NSCLC at diagnosis**	945 (79.9)	506 (79.7)	362 (80.1)	77 (81.1)
**NSCLC histology, *n*** [a]	1130	609	430	91
Nonsquamous	698 (61.8)	383 (62.9)	270 (62.8)	45 (49.5)
Squamous	360 (31.9)	188 (30.9)	129 (30.0)	43 (47.3)
Other	72 (6.4)	38 (6.2)	31 (7.2)	3 (3.3)
**Brain metastasis**	194 (16.4)	96 (15.1)	92 (20.4)	6 (6.3)
Pretreated [b]	126 (64.9)	56 (58.3)	66 (71.7)	4 (66.7)
**Liver metastasis**	96 (8.1)	50 (7.9)	36 (8.0)	10 (10.5)
**Bone metastasis**	296 (25.0)	162 (25.5)	120 (26.5)	14 (14.7)
**History of lung surgery**	203 (17.2)	121 (19.1)	67 (14.8)	15 (15.8)
**Prior radiation therapy**	94 (8.0)	45 (7.1)	41 (9.1)	8 (8.4)
**Prior chemoradiation**	38 (3.2)	18 (2.8)	18 (4.0)	2 (2.1)

Data are *n* (%) unless otherwise indicated. Percentages may not add up to 100% because of rounding. [a] Percentages are of known totals. [b] Brain metastases treated before initiation of first-line therapy. ECOG PS, Eastern Cooperative Oncology Group performance status; ICI, immune checkpoint inhibitor.

Table S3. Real-world treatment outcomes for first-line regimens.

	**Platinum** *n* **= 635**	**ICI Monotherapy** *n* **= 452**	**Nonplatinum** *n* **= 95**
**First-line rwToT, *n***	635	452	95
Events, *n* (%)	599 (94.3)	428 (94.7)	87 (91.6)
Censored, *n* (%)	36 (5.7)	24 (5.3)	8 (8.4)
Median rwToT (95% CI), mo	3.0 (2.8–3.3)	5.5 (4.4–6.7)	2.2 (1.2–3.2)
On-treatment rate, % (95% CI) [a]			
At 6 months	18.6 (15.6–21.9)	48.8 (44.0–53.5)	17.7 (10.5–26.4)
At 12 months	5.9 (4.2–8.0)	25.6 (21.5–29.8)	3.5 (0.9–9.1)
At 18 months	1.2 (0.5–2.3)	12.1 (9.2–15.4)	2.4 (0.5–7.4)
**rwTTNT, *n***	634	452	95
Events, *n* (%)	404 (63.7)	167 (36.9)	44 (46.3)
Censored, *n* (%)	230 (36.3)	285 (63.1)	51 (53.7)
Median rwTTNT (95% CI), mo	6.3 (5.9–6.9)	18.3 (14.0–NR)	6.7 (4.3–9.4)
rwTTNT rate, % (95% CI) [a]			
At 6 months	53.6 (49.3–57.7)	77.2 (72.6–81.1)	53.2 (40.9–64.0)
At 12 months	25.1 (21.2–29.3)	58.9 (53.4–63.9)	33.7 (21.5–46.4)
At 24 months	13.8 (9.1–19.4)	46.9 (39.3–54.2)	n/a

[a] rwTTNT was determined only by first-line regimen. The rwTTNT rate represents the percentage of patients who had not initiated a second-line regimen at 6, 12, and 24 months. n/a, not assessed; NR, not reached; rw, real-world; ToT, time on treatment; TTNT, time to next treatment.

Table S4. Treatment sequence overall and by tumor histology.

Real-World Treatment Sequence, *n* (%)	
**All patients**	**(*n* = 1182)**
1L Platinum doublet; 2L none; 3L none	230 (19.5)
1L Platinum doublet; 2L ICI - Monotherapy; 3L ICI - Monotherapy	6 (0.5)
1L Platinum doublet; 2L ICI - Monotherapy; 3L Nonplatinum cytotoxic agent	94 (8.0)
1L Platinum doublet; 2L ICI - Monotherapy; 3L Platinum doublet	10 (0.8)
1L Platinum doublet; 2L ICI - Monotherapy; 3L none	149 (12.6)
1L Platinum doublet; 2L Platinum doublet; 3L ICI - Monotherapy	15 (1.3)
1L Platinum doublet; 2L Platinum doublet; 3L Nonplatinum cytotoxic agent	9 (0.8)
1L Platinum doublet; 2L Platinum doublet; 3L Other	1 (0.1)
1L Platinum doublet; 2L Platinum doublet; 3L none	12 (1.0)
1L Platinum doublet; 2L Nonplatinum cytotoxic agent; 3L ICI - Monotherapy	38 (3.2)
1L Platinum doublet; 2L Nonplatinum cytotoxic agent; 3L Nonplatinum cytotoxic agent	16 (1.4)
1L Platinum doublet; 2L Nonplatinum cytotoxic agent; 3L Platinum doublet	1 (0.1)
1L Platinum doublet; 2L Nonplatinum cytotoxic agent; 3L none	53 (4.5)
1L Platinum doublet; 2L Tyrosine Kinase Inhibitors; 3L none	1 (0.1)
1L ICI - Monotherapy; 2L none; 3L none	285 (24.1)
1L ICI - Monotherapy; 2L ICI - Monotherapy; 3L Nonplatinum cytotoxic agent	1 (0.1)
1L ICI - Monotherapy; 2L ICI - Monotherapy; 3L none	2 (0.2)
1L ICI - Monotherapy; 2L ICI - Combination with chemotherapy; 3L none	1 (0.1)
1L ICI - Monotherapy; 2L Platinum doublet; 3L ICI - Monotherapy	10 (0.8)
1L ICI - Monotherapy; 2L Platinum doublet; 3L Nonplatinum cytotoxic agent	42 (3.6)
1L ICI - Monotherapy; 2L Platinum doublet; 3L Other	1 (0.1)
1L ICI - Monotherapy; 2L Platinum doublet; 3L Platinum doublet	6 (0.5)
1L ICI - Monotherapy; 2L Platinum doublet; 3L Tyrosine Kinase Inhibitors	2 (0.2)
1L ICI - Monotherapy; 2L Platinum doublet; 3L none	65 (5.5)
1L ICI - Monotherapy; 2L Nonplatinum cytotoxic agent; 3L ICI - Monotherapy	4 (0.3)
1L ICI - Monotherapy; 2L Nonplatinum cytotoxic agent; 3L Nonplatinum cytotoxic agent	10 (0.8)
1L ICI - Monotherapy; 2L Nonplatinum cytotoxic agent; 3L Platinum doublet	1 (0.1)
1L ICI - Monotherapy; 2L Nonplatinum cytotoxic agent; 3L none	21 (1.8)
1L ICI - Monotherapy; 2L Other; 3L none	1 (0.1)
1L Nonplatinum cytotoxic agent; 2L none; 3L none	51 (4.3)
1L Nonplatinum cytotoxic agent; 2L ICI - Monotherapy; 3L ICI - Monotherapy	1 (0.1)
1L Nonplatinum cytotoxic agent; 2L ICI - Monotherapy; 3L Nonplatinum cytotoxic agent	6 (0.5)
1L Nonplatinum cytotoxic agent; 2L ICI - Monotherapy; 3L none	23 (1.9)
1L Nonplatinum cytotoxic agent; 2L Nonplatinum cytotoxic agent; 3L ICI - Monotherapy	1 (0.1)
1L Nonplatinum cytotoxic agent; 2L Nonplatinum cytotoxic agent; 3L Nonplatinum cytotoxic agent	3 (0.3)
1L Nonplatinum cytotoxic agent; 2L Nonplatinum cytotoxic agent; 3L none	10 (0.8)
**Nonsquamous histology**	**(*n* = 698)**
1L Platinum doublet; 2L none; 3L none	146 (20.9)
1L Platinum doublet; 2L ICI - Monotherapy; 3L ICI - Monotherapy	2 (0.3)
1L Platinum doublet; 2L ICI - Monotherapy; 3L Nonplatinum cytotoxic agent	55 (7.9)
1L Platinum doublet; 2L ICI - Monotherapy; 3L Platinum doublet	4 (0.6)
1L Platinum doublet; 2L ICI - Monotherapy; 3L none	81 (11.6)
1L Platinum doublet; 2L Platinum doublet; 3L ICI - Monotherapy	7 (1.0)
1L Platinum doublet; 2L Platinum doublet; 3L Nonplatinum cytotoxic agent	8 (1.1)
1L Platinum doublet; 2L Platinum doublet; 3L Other	1 (0.1)
1L Platinum doublet; 2L Platinum doublet; 3L none	6 (0.9)
1L Platinum doublet; 2L Nonplatinum cytotoxic agent; 3L ICI - Monotherapy	30 (4.3)
1L Platinum doublet; 2L Nonplatinum cytotoxic agent; 3L Nonplatinum cytotoxic agent	9 (1.3)

1L Platinum doublet; 2L Nonplatinum cytotoxic agent; 3L none	33 (4.7)
1L Platinum doublet; 2L Tyrosine Kinase Inhibitors; 3L none	1 (0.1)
1L ICI - Monotherapy; 2L none; 3L none	167 (23.9)
1L ICI - Monotherapy; 2L ICI - Monotherapy; 3L Nonplatinum cytotoxic agent	1 (0.1)
1L ICI - Monotherapy; 2L ICI - Monotherapy; 3L none	1 (0.1)
1L ICI - Monotherapy; 2L ICI - Combination with chemotherapy; 3L none	1 (0.1)
1L ICI - Monotherapy; 2L Platinum doublet; 3L ICI - Monotherapy	7 (1.0)
1L ICI - Monotherapy; 2L Platinum doublet; 3L Nonplatinum cytotoxic agent	28 (4.0)
1L ICI - Monotherapy; 2L Platinum doublet; 3L Other	1 (0.1)
1L ICI - Monotherapy; 2L Platinum doublet; 3L Platinum doublet	5 (0.7)
1L ICI - Monotherapy; 2L Platinum doublet; 3L Tyrosine Kinase Inhibitors	2 (0.3)
1L ICI - Monotherapy; 2L Platinum doublet; 3L none	39 (5.6)
1L ICI - Monotherapy; 2L Nonplatinum cytotoxic agent; 3L ICI - Monotherapy	3 (0.4)
1L ICI - Monotherapy; 2L Nonplatinum cytotoxic agent; 3L Nonplatinum cytotoxic agent	6 (0.9)
1L ICI - Monotherapy; 2L Nonplatinum cytotoxic agent; 3L Platinum doublet	1 (0.1)
1L ICI - Monotherapy; 2L Nonplatinum cytotoxic agent; 3L none	7 (1.0)
1L ICI - Monotherapy; 2L Other; 3L none	1 (0.1)
1L Nonplatinum cytotoxic agent; 2L none; 3L none	26 (3.7)
1L Nonplatinum cytotoxic agent; 2L ICI - Monotherapy; 3L Nonplatinum cytotoxic agent	4 (0.6)
1L Nonplatinum cytotoxic agent; 2L ICI - Monotherapy; 3L none	9 (1.3)
1L Nonplatinum cytotoxic agent; 2L Nonplatinum cytotoxic agent; 3L ICI - Monotherapy	1 (0.1)
1L Nonplatinum cytotoxic agent; 2L Nonplatinum cytotoxic agent; 3L none	5 (0.7)
**Squamous histology**	**(*n* = 360)**
1L Platinum doublet; 2L none; 3L none	66 (18.3)
1L Platinum doublet; 2L ICI - Monotherapy; 3L ICI - Monotherapy	4 (1.1)
1L Platinum doublet; 2L ICI - Monotherapy; 3L Nonplatinum cytotoxic agent	29 (8.1)
1L Platinum doublet; 2L ICI - Monotherapy; 3L Platinum doublet	5 (1.4)
1L Platinum doublet; 2L ICI - Monotherapy; 3L none	50 (13.9)
1L Platinum doublet; 2L Platinum doublet; 3L ICI - Monotherapy	7 (1.9)
1L Platinum doublet; 2L Platinum doublet; 3L none	3 (0.8)
1L Platinum doublet; 2L Nonplatinum cytotoxic agent; 3L ICI - Monotherapy	6 (1.7)
1L Platinum doublet; 2L Nonplatinum cytotoxic agent; 3L Nonplatinum cytotoxic agent	4 (1.1)
1L Platinum doublet; 2L Nonplatinum cytotoxic agent; 3L none	14 (3.9)
1L ICI - Monotherapy; 2L none; 3L none	80 (22.2)
1L ICI - Monotherapy; 2L Platinum doublet; 3L ICI - Monotherapy	2 (0.6)
1L ICI - Monotherapy; 2L Platinum doublet; 3L Nonplatinum cytotoxic agent	11 (3.1)
1L ICI - Monotherapy; 2L Platinum doublet; 3L none	20 (5.6)
1L ICI - Monotherapy; 2L Nonplatinum cytotoxic agent; 3L ICI - Monotherapy	1 (0.3)
1L ICI - Monotherapy; 2L Nonplatinum cytotoxic agent; 3L Nonplatinum cytotoxic agent	3 (0.8)
1L ICI - Monotherapy; 2L Nonplatinum cytotoxic agent; 3L none	12 (3.3)
1L Nonplatinum cytotoxic agent; 2L none; 3L none	21 (5.8)
1L Nonplatinum cytotoxic agent; 2L ICI - Monotherapy; 3L ICI - Monotherapy	1 (0.3)
1L Nonplatinum cytotoxic agent; 2L ICI - Monotherapy; 3L Nonplatinum cytotoxic agent	2 (0.6)
1L Nonplatinum cytotoxic agent; 2L ICI - Monotherapy; 3L none	12 (3.3)
1L Nonplatinum cytotoxic agent; 2L Nonplatinum cytotoxic agent; 3L Nonplatinum cytotoxic agent	2 (0.6)
1L Nonplatinum cytotoxic agent; 2L Nonplatinum cytotoxic agent; 3L none	5 (1.4)

1L, 2L, 3L, first-, second-, third-line therapy; ICI, immune checkpoint inhibitor.

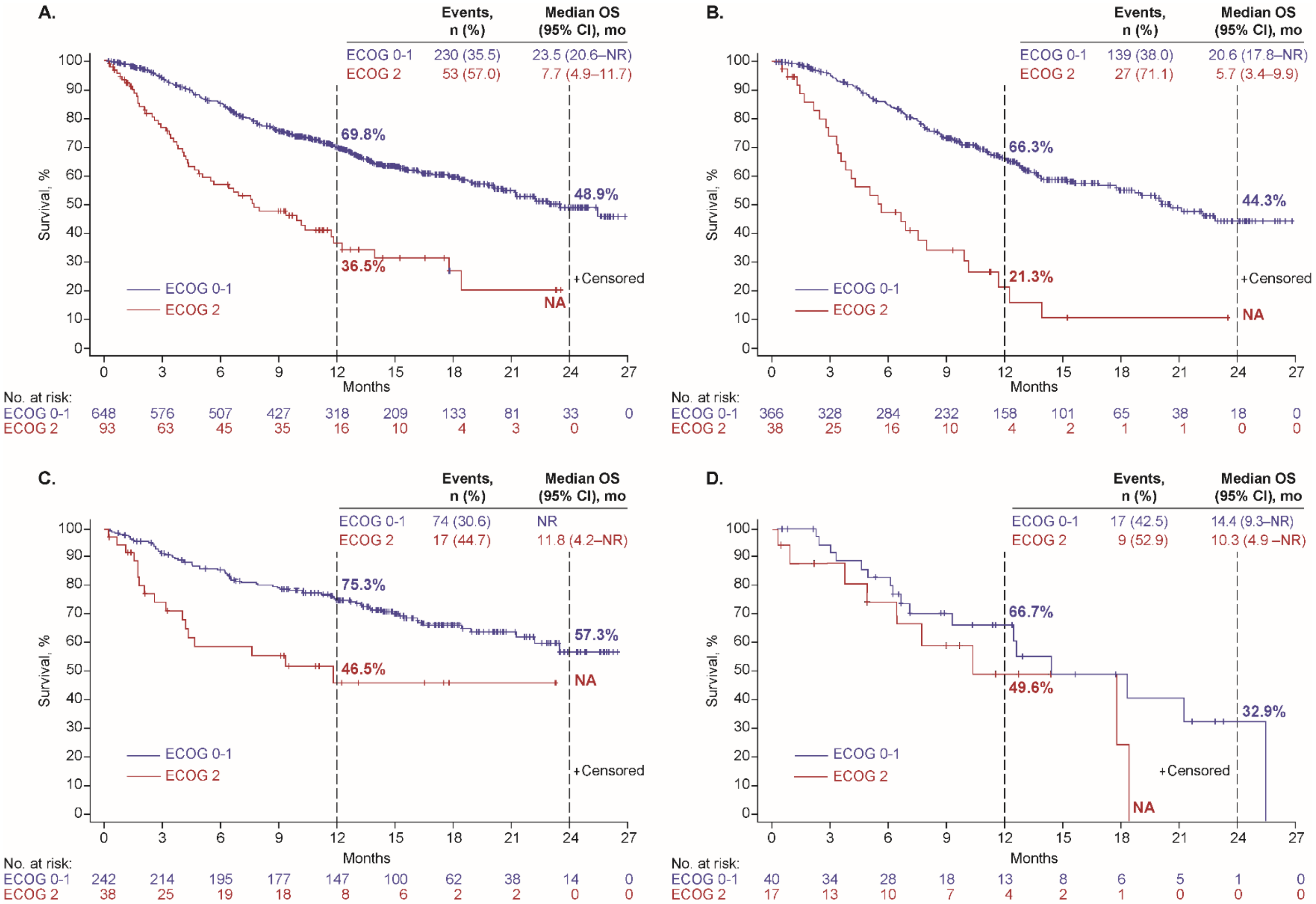

Figure S1. Overall survival by ECOG performance status among patients who received first-line therapy (**A**) overall and with (**B**) platinum doublet, (**C**) ICI monotherapy, and (**D**) nonplatinum cytotoxic regimen.

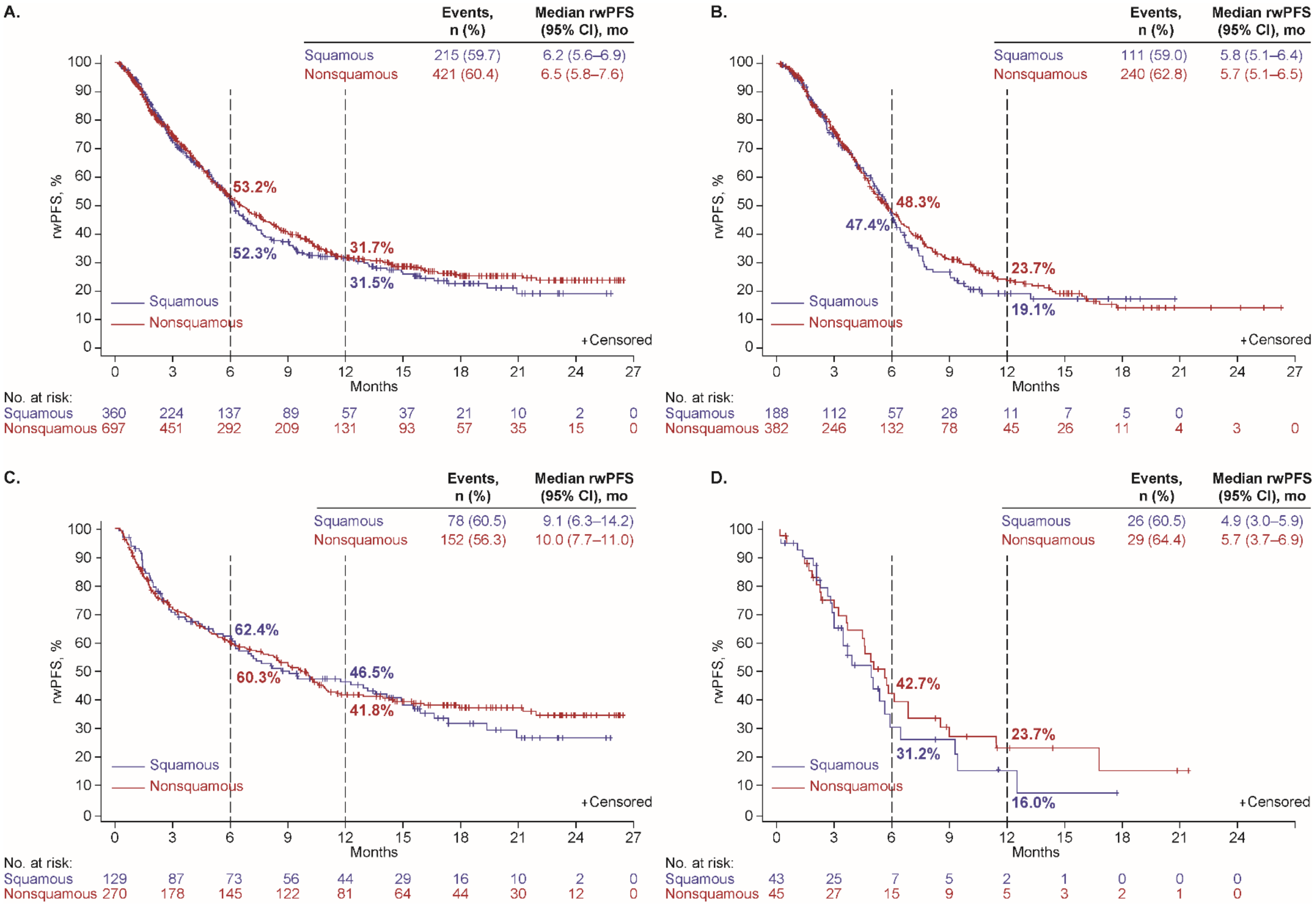

Figure S2. Real-world progression-free survival (rwPFS) by tumor histology among patients who received first-line therapy (**A**) overall and with (**B**) platinum doublet, (**C**) ICI monotherapy, and (**D**) nonplatinum cytotoxic regimen.

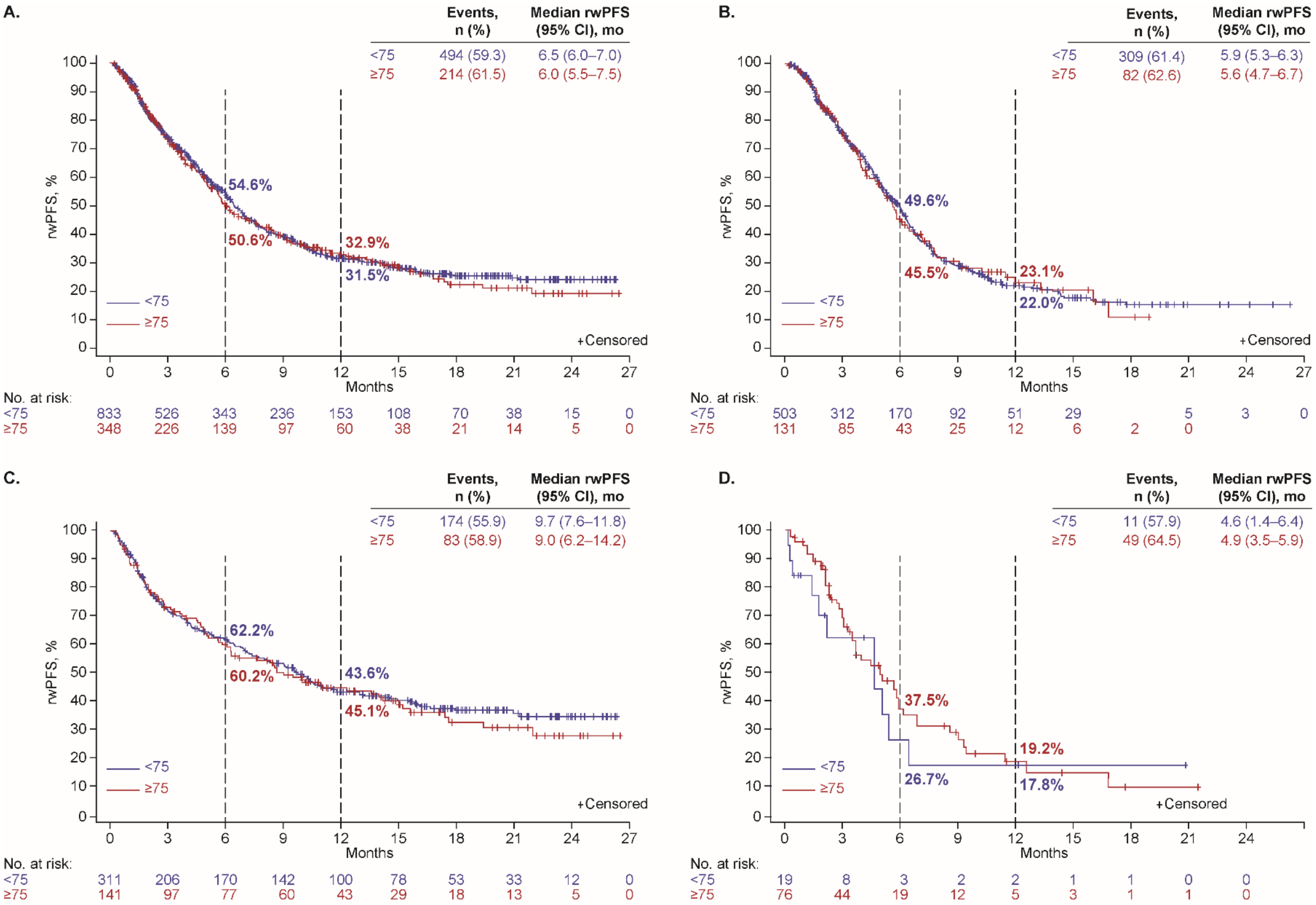

Figure S3. Real-world progression-free survival (rwPFS) by age group among patients who received first-line therapy (**A**) overall and with (**B**) platinum doublet, (**C**) ICI monotherapy, and (**D**) nonplatinum cytotoxic regimen.

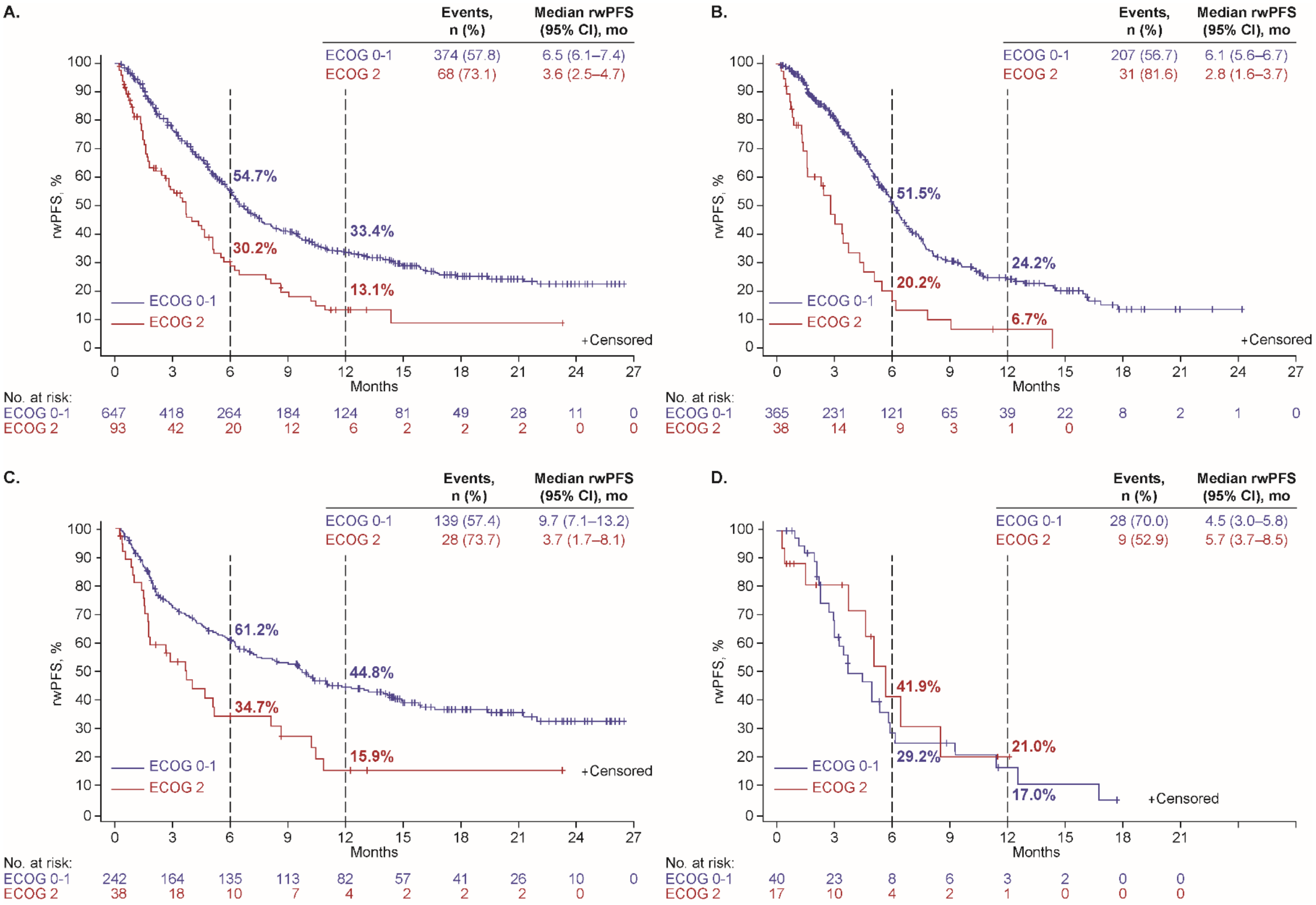

Figure S4. Real-world progression-free survival (rwPFS) by ECOG PS among patients who received first-line therapy (**A**) overall and with (**B**) platinum doublet, (**C**) ICI monotherapy, and (**D**) nonplatinum cytotoxic regimen.

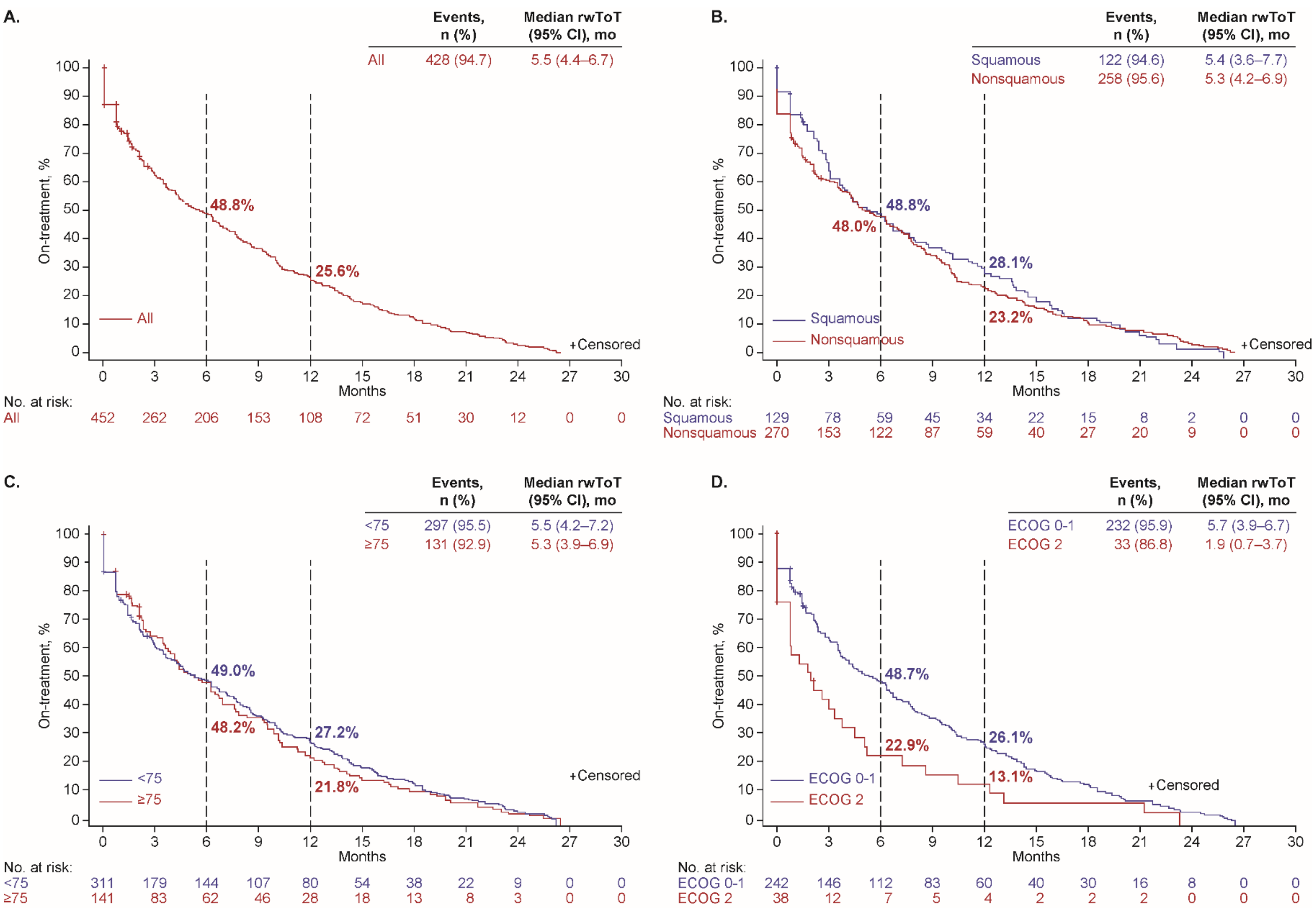

Figure S5. Real-world time on treatment for patients who received first-line ICI monotherapy (**A**) overall; (**B**) by histology, (**C**) by age group, and (**D**) by ECOG PS.
